# Spigelian Hernia Unveiled: A Case Report of Unexpectedly Extended Hernial Sac and Successful Surgical Intervention

**DOI:** 10.1002/ccr3.9605

**Published:** 2024-11-26

**Authors:** Marah Mansour, Yaman Saiouf, Karim Gharib, Dani Deeb, Mohammad Yaman Almokdad

**Affiliations:** ^1^ Faculty of Medicine Tartous University Tartous Syrian Arab Republic; ^2^ Division of Colon and Rectal Surgery Department of Surgery Mayo Clinic Rochester Minnesota USA; ^3^ Faculty of Medicine Damascus University Damascus Syrian Arab Republic; ^4^ Faculty of Medicine Albaath University Homs Syrian Arab Republic; ^5^ Department of General Surgery Alassad University Hospital Damascus Syria

**Keywords:** case report, inguinal hernia, open surgery, Spigelian hernia

## Abstract

Spigelian hernia is a rare type of abdominal hernia; diagnosis is primarily through computed tomography, which is crucial for accurate diagnosis and planning early surgical intervention to preserve the herniated sac's contents.

**Abbreviations**
CT: computed tomography
SH: spigelian hernia
US: ultrasound

## 1 Introduction

Spigelian hernia (SH) is an uncommon defect in the Spigelian fascia, centralized laterally by the semilunar line and medially by the rectus muscle [1, 2, 3, 4, 5]. It is also defined as an automatic lateral ventral hernia in the semilunar line [1, 3, 4, 6, 7]. In 1764, a lateral ventral hernia located in the semilunar line was described by Klinkosch [2, 7]. The incidence of SH represents 1%–2% of all abdominal hernias [2, 8]. SH has been described in people of all ages, including neonates [9]. The incidence is higher in males at a young age, while females are more affected in individuals over 60 years old [2, 8]. There are no specific causes for SH, but the association with respiratory diseases, multiple pregnancies, and obesity with fast weight loss was described [2]. Patients suffer from localized pain that sometimes becomes diffuse and aggravating [2, 7, 10, 11]. SH is crucially difficult to diagnose clinically because of its unclear symptoms and signs and its localization between muscular layers [10, 12]. Radiological examinations such as ultrasound (US), computed tomography (CT), and magnetic resonance imaging are necessary to confirm an SH diagnosis [7, 10, 12, 13]. CT is the most useful diagnostic tool [13]. The classic treatment of choice is open surgery. Recently, laparoscopic techniques have been used for treating SH, with good outcomes for both techniques [3, 4, 8, 12, 13, 14]. In this case, we documented a SH that was managed by an open abdominal incision without complications.

## 2 Case History/Examination

A 54‐year‐old female was admitted to the Department of General Surgery with constantly increasing pain in her left flank for 4 days. The patient had localized deep pain, worsened with cough. The last defecation was 3 days ago. Medical history included a C‐section, a right mastectomy with 6 doses of chemotherapy 13 years ago, and smoking tobacco (14 packs/year). On physical examination, the abdomen was soft, with tenderness, along with a protrusion in the left inguinal region. The cardiovascular examination was normal.

## 3 Differential Diagnosis, Investigations, and Treatment

**FIGURE 1:**
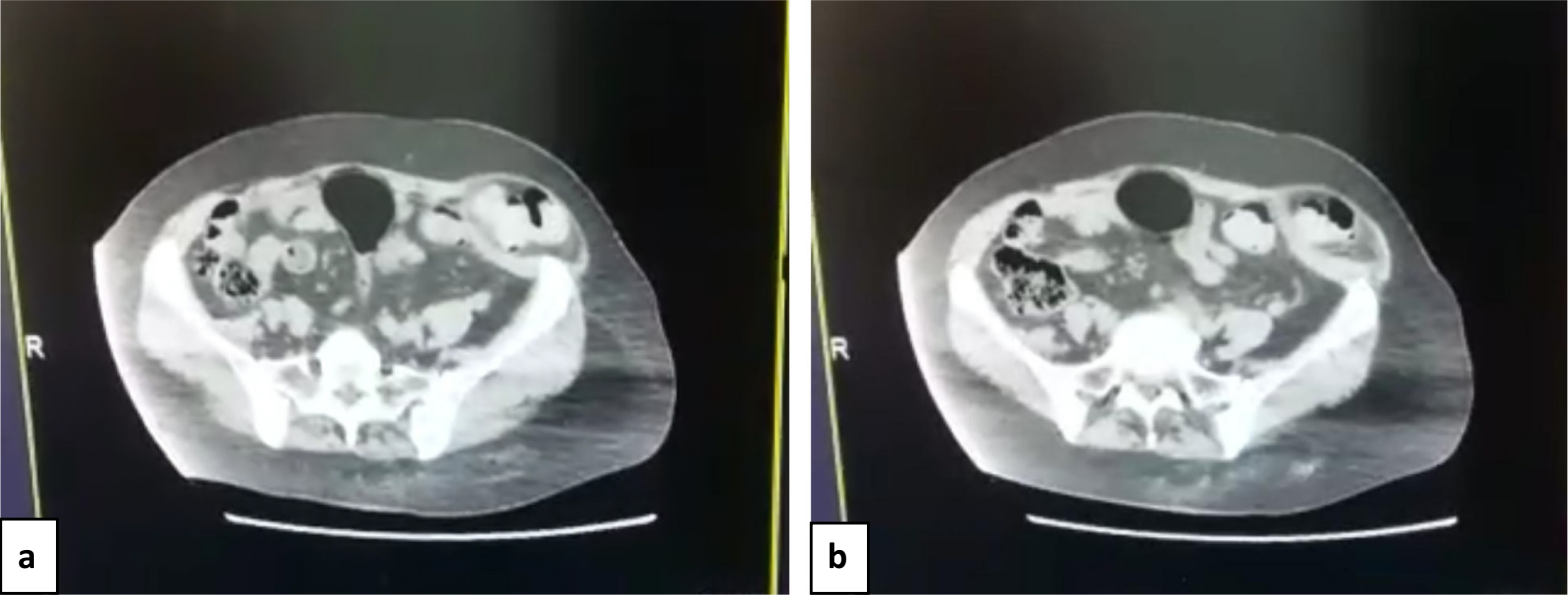
The CT‐Scan revealed a left inguinal hernia measuring 8.5 mm, containing loops of the small and large intestine, the end of the descending colon, and sigmoid colon, with suspicion of a sigmoid volvulus.

**FIGURE 2:**
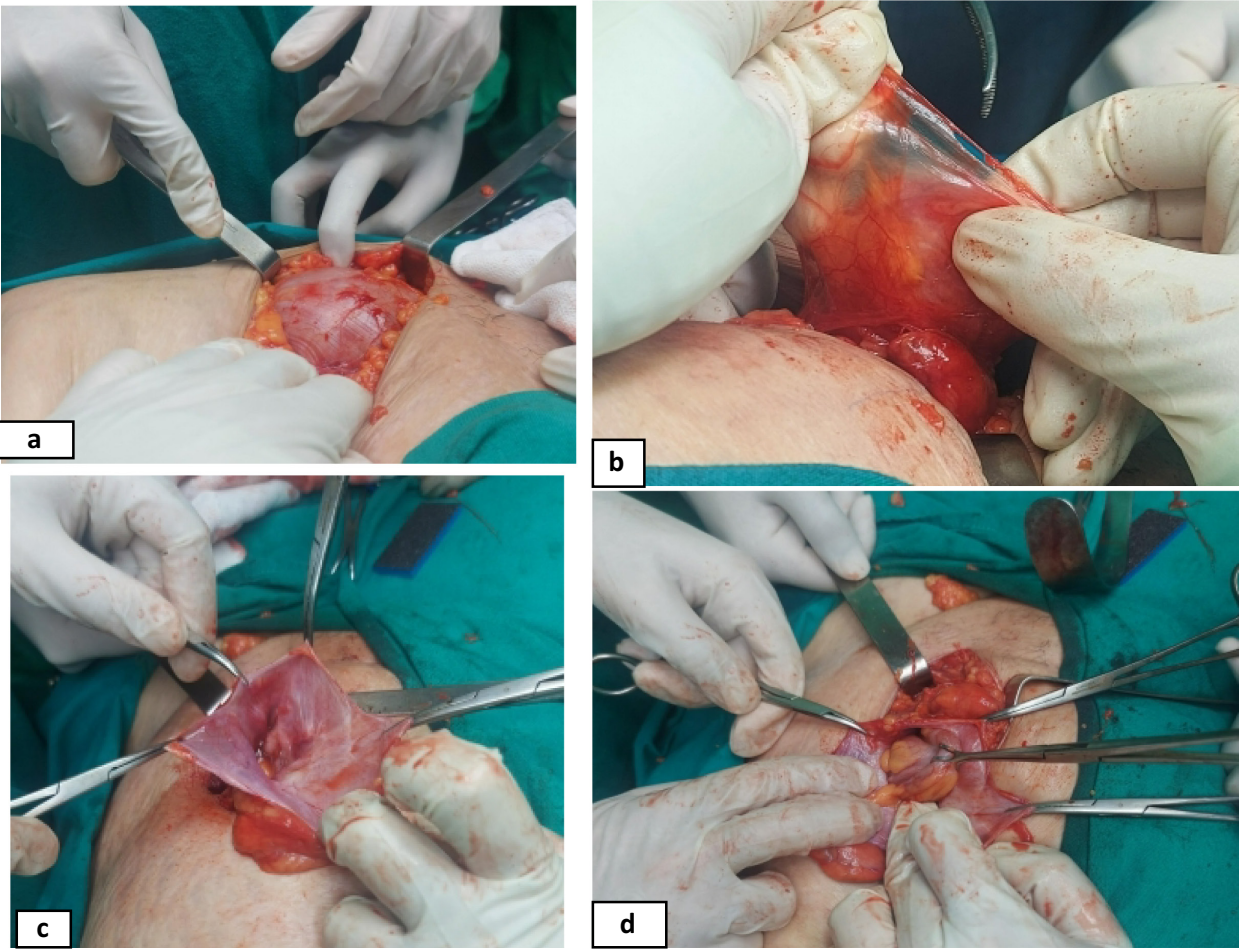
(a) Incision made through the epidermis and subcutaneous layers revealing the prominent aponeurosis of the external oblique muscle. (b) Hernia sac isolated for further examination. (c) Hernial sac exposed for reduction and anatomical restoration. (d) The contents of the hernia sac without complications.

**FIGURE 3:**
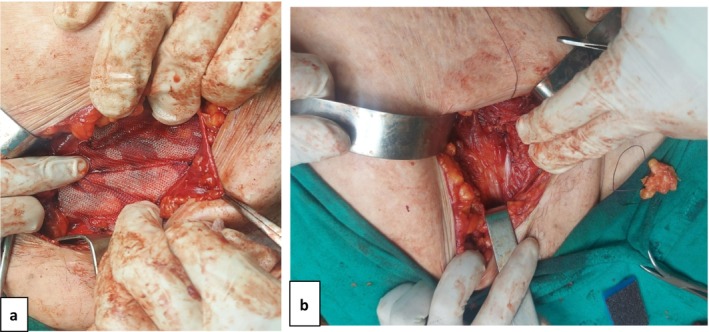
(a) Placement of the mesh before closure of the surgical incision. (b) Subcutaneous third space (SH) fixed in place after the procedure.

Routine blood tests were within normal limits. CT and US confirmed the diagnosis of a left inguinal hernia measuring 8.5 mm, which included loops of small and large intestines, the end of the descending colon, and the sigmoid colon (Figure 1a,b). In addition, sigmoid volvulus was suspected. Fecal impactions were found in the transverse and ascending colons. The surgery was performed under general anesthesia. A left paramedian incision was made, and a SH was discovered (Figure 2a). The sac was opened (Figure 2b–d), the vitality of the contents (sigmoid colon) was examined, and a correction was made by returning it to the abdominal cavity. The sac was also tied and returned. The inner wall (obliquus internus and transversus abdominis) was closed. A patch and section were placed (polypropylene mesh 10 × 10 cm), and the aponeurosis of the visible oblique muscle was then closed (Figure 3a,b). The polypropylene mesh is positioned over the hernia defect, ensuring adequate coverage and fixation to the surrounding tissues using non‐absorbable sutures. This step aims to reinforce the weakened abdominal wall and prevent recurrence of the hernia postoperatively.

The skin was closed in layers, and a bandage was applied.

## 4 Outcome and Follow‐Up

Post‐operatively, the patient was prescribed antibiotics (amoxicillin/clavulanic acid 1 g BID: 7 days) and anti‐edema medications (Bromelain 200 BID: 7 days) with an abdominal corset for 2 months. The goal of compression garments is to provide compression to the skin and soft tissues, alleviating pain, reducing swelling, and improving overall healing. Additionally, sudden movements like coughing, laughing, and sneezing can be uncomfortable. Within a 2‐month follow‐up, the patient was in good condition without any recurrence.

## 5 Discussion

SH occurs in the Spigelian fascia between the rectus muscle in the medial and the semilunar line in the lateral [1, 2, 3, 4, 5]. SH is defined as a lateral spontaneous abdominal hernia in the semicircular line [1, 3, 4, 6, 7]. The semilunar line was first described by Adriaan van der Spiegel in 1645 [2, 7]. Its incidence is 1%–2% of abdominal hernias, and it is also rarely coincidental [2, 8]. Spiegel can be seen in any age group, but it often occurs in women over the age of 60 [2, 7]. Its etiology is not clear, as obesity with rapid weight loss, multiple births, pregnancy, and respiratory diseases are all considered possible causes [2, 15]. Most patients present with bulges or pain localized in the hernia area [2, 7, 10, 11, 14]. SH is difficult to diagnose because of its location between the muscle layers and its lack of distinct symptoms [10, 12]. US and CT scans are the most beneficial diagnostic tools [7, 10, 12, 13].

US is typically the initial evaluation method used to assess suspected inguinal hernias and is considered a safe screening tool. However, its accuracy for differentiating inguinal hernias can vary. In cases where uncertainty remains, CT may be employed because of its higher accuracy, although caution is advised due to radiation exposure risks [12].

CT allows for precise localization of the hernia, making it a valuable diagnostic tool for determining the most appropriate treatment and assessing potential complications [13]. Herniography is another option that can provide a detailed assessment in cases where the diagnosis remains uncertain after US [12]. Given the potential for false negatives in radiological investigations, some surgeons may opt for exploratory laparoscopy, which is considered the definitive diagnostic method for such cases [12].

The SH treatment is surgery by opening the abdomen; however, it has begun to be replaced by laparoscopic surgery, with good results for both techniques [3, 4, 8, 12, 13, 14]. To repair SHs, laparoscopy can be safely and effectively used. This results in less morbidity and shorter hospitalizations compared with open procedures, as recent evidence has demonstrated [3, 15]. If untreated, the hernia may become strangulated, which may lead to bowel necrosis, bowel obstruction, or both, and perforation [15]. In our case, the patient was a 54‐year‐old female who presented with increasing pain that was localized in the left flank area and a lack of defecation.

On clinical examination, the abdomen was soft and tender, with swelling in the left inguinal region. The inguinal swelling could be due to an extension of the Spigelian hernia sac into the inguinal region. This can happen if the hernia sac follows the path of least resistance and extends through the inguinal canal.

The primary method of diagnosis was CT scan, as the radiograph showed misleading results, and the radiology report indicated the possible presence of an inguinal hernia due to its poor positioning (Figure 1a,b).

The contents of the hernia sac were disrupted and extended an unexpected distance away from the hernia orifice toward the inguinal side down and laterally. A Spigelian hernia occurs through the Spigelian fascia, which is located between the rectus abdominis muscle and the semilunar line. If the hernia sac extends into the inguinal region, it could be due to the hernia sac following the path of least resistance, potentially pushing through weakened areas of the abdominal wall. The hernia sac would typically travel through the Spigelian fascia, then potentially through the transversalis fascia, and into the inguinal canal if it extends that far.

The surgery involved opening the abdomen and making a vertical incision at the most common location of the hernia (Figure 2a). Hernial contents were abundant, with intestinal viability and sigmoid torsion. It was saved before outright suffocation of the contents of the hernia sac occurred (Figure 2b–d). The hernia was properly managed by returning it into the abdominal cavity without undergoing unnecessary surgeries (Figure 3a,b). Recovery was uneventful; the patient was in good condition, with no recurrence.

## 6 Conclusion

This report emphasizes the necessity of focusing radiographic imaging on inguinal hernias, along with the various contents of hernia sacs and their extent of involvement. The aim of this report is to highlight the importance of considering inguinal hernia as a potential diagnosis, which contributes to appropriate management and minimizes the risk of complications. The results of CT have demonstrated its effectiveness as a diagnostic tool for inguinal hernia in this case.

## Author Contributions

**Marah Mansour:** formal analysis, project administration, writing – original draft, writing – review and editing. **Yaman Saiouf:** formal analysis, methodology, writing – original draft, writing – review and editing. **Karim Gharib:** formal analysis, writing – original draft, writing – review and editing. **Dani Deeb:** formal analysis, writing – original draft, writing – review and editing. **Mohammad Yaman Almokdad:** data curation, investigation, supervision.

## Consent

Written informed consent was obtained from the patient for publishing this case report and any accompanying images. A copy of the written consent is available for review by the Editor‐in‐Chief of this journal on request.

## Data Availability

All data (of the patient) generated during this study are included in this published article and its supplementary information files.
